# The IL-10 polarized cytokine pattern in innate and adaptive immunity cells contribute to the development of FVIII inhibitors

**DOI:** 10.1186/s12878-014-0019-8

**Published:** 2015-01-16

**Authors:** Amanda CO Silveira, Marcio AP Santana, Isabella G Ribeiro, Daniel G Chaves, Olindo A Martins-Filho

**Affiliations:** Laboratório de Biomarcadores de Diagnóstico e Monitoração, Centro de Pesquisas René Rachou, FIOCRUZ/MG, Belo Horizonte, Minas Gerais Brazil; Serviço de Pesquisa, Fundação Hemominas, Belo Horizonte, Minas Gerais Brazil

**Keywords:** Immune regulation, Intracellular cytokine staining, Cytokine profile, FVIII inhibitors, Hemophilia A

## Abstract

**Background:**

Hemophilia A (HA) is an X-linked inherited bleeding disorder, resulting from a qualitative or quantitative deficiency of clotting factor VIII (FVIII). Antibodies against FVIII, also called inhibitors, block the procoagulant activity of FVIII; thus, impairing hemostatic activity in patients with HA. The exact mechanism underlying the immunological events behind the development of inhibitors remains unknown. This study aimed to understand immune response to FVIII in patients with HA who were either positive [HAα-FVIII(+)] or negative [HAα-FVIII(−)] for inhibitors.

**Methods:**

Cytokine profiles [interferon-γ (IFN − γ), tumor necrosis factor-α (TNF-α), interleukin-4 (IL-4), IL-5, and IL-10] of innate and adaptive immune cells present in the peripheral blood of participants were characterized.

**Results:**

Presence of inhibitors was significantly associated with decreased frequencies of TNF-α-positive monocytes and neutrophils, IL-5-positive monocytes, IL-4-positive neutrophils, and increased frequencies of IL-10-positive neutrophils and T cells. T cells from HAα-FVIII(−) patients expressed increased levels of almost all cytokines. In contrast, HAα-FVIII(+) patients showed lower levels of all cytokines in CD4^+^ and CD8^+^ T cells, except IL-10. B cells from HAα-FVIII(−) patients expressed increased levels of IL-4 while those from HAα-FVIII(+) patients expressed increased levels of IL-10.

**Conclusions:**

The global cytokine profiles of innate and adaptive immune cells showed an anti-inflammatory/regulatory pattern in HAα-FVIII(+) patients and a mixed pattern, with a bias toward inflammatory cytokine profile, in HAα-FVIII(−) patients. The occurrence of these profiles seems to be associated with presence FVIII inhibitors.

## Background

Hemophilia A (HA) is an X-linked inherited bleeding disorder resulting from a qualitative or quantitative deficiency of clotting factor VIII (FVIII) [1]. HA is classified as mild, moderate, or severe based on the degree of FVIII residual activity [2]. Treatment of patients with HA involves replacement therapy with plasma-derived FVIII (pdFVIII) or recombinant FVIII [3]. A major clinical complication observed during replacement therapy is the development of antibodies against FVIII (called inhibitors) that block its procoagulant activity. Approximately 10%–15% patients with HA and 25%–30% patients with severe HA develop inhibitors [4]. Patient’s age at the time of the first exposure to replacement therapy and type and frequency of FVIII exposure are risk factors for inhibitor formation; in addition, mutations in the gene encoding FVIII and variations in the immune system are important risk factors for inhibitor formation [5]. Anti-FVIII antibodies are immunoglobulin G (IgG) antibodies, mainly IgG4; in some cases, IgG1 and IgG2 can also be detected [6,7].

Type 1 cells have been established to play a role in the initial immune response to FVIII, and type 2 cells act in the development of strong inhibitor production. CD4^+^ T cells are important for the production of inhibitors because they secrete both proinflammatory and anti-inflammatory/regulatory cytokines [8]. Studies have described that polymorphisms in genes encoding cytokines such as TNF-α and IL-10 greatly affect inhibitor production [5,9-11]. Several studies have focused on immune response in patients with HA to elucidate the mechanisms underlying inhibitor production. We observed that the global cytokine profiles of innate and adaptive immune cells showed a major anti-inflammatory/regulatory pattern in patients with HA who were positive for inhibitors [HAα-FVIII(+)] and showed a mixed pattern, with a bias toward an inflammatory cytokine profile, in patients who were negative for inhibitors [HAα-FVIII(−)]. In addition, we proposed that these cytokine profile patterns may be the key elements in the production of distinct subclasses of anti-FVIII antibodies [12-14]. To understand immune response to FVIII, we characterized the cytokine patterns of peripheral blood leukocytes from whole blood samples of healthy blood donors, HAα-FVIII(+) patients, and HAα-FVIII(−) patients. In addition, we examined the differential synthesis of proinflammatory (IFN-γ and TNF-α) and anti-inflammatory/regulatory (IL-4, IL-5, and IL-10) cytokines in innate (neutrophils and monocytes) and adaptive (CD4^+^ and CD8^+^ T and B cells) immune cells.

## Methods

### Study population and sample collection

This case–control study included 85 subjects who were classified into three groups: (1) healthy blood donors (BDs; *n* = 30; mean age, 31.6 ± 12.8 years), (2) patients with HA without history of inhibitors (HAα-FVIII(−); *n* = 30; mean age, 27.6 ± 16.6 years), and (3) patients with HA who had inhibitors (HAα-FVIII(+); *n* = 25; mean age, 21.9 ± 13.8 years; mean anti-FVIII inhibitor level at the time of blood collection, 11.0 UB/mL). All the patients received on-demand (episodic) treatment and were paired by gender and age. Table 1 summarizes the main characteristics of HAα-FVIII(+) patients. Heparinized blood samples from all the subjects were collected in vacutainer tubes (BD Pharmingen, San Diego, CA, USA). All the study subjects visited Fundação Hemominas, Minas Gerais, Brazil. This study was approved by the ethics committee of the Hemominas Foundation and by the National Commission of Ethics in Research, Brazil. Written informed consent for participation in the study was obtained from participants or their parent or their guardian where applicable. Patients with HA are frequently examined at the Fundação Hemominas to determine the presence of inhibitors. HAα-FVIII(+) patients were selected based on their positive history of inhibitors. Median time between the first inhibitor production and the time of sampling was 170.8 months (range, 10.9–241.0 months). Any results above 0.6 UB/mL were considered positive for the presence of inhibitors. Patients were selected by carefully analyzing their medical records. Individuals with a history of transient inhibitor production were excluded from the study. All patients with HA did not receive FVIII infusions for 30 days before the date of blood collection to avoid any influence due to a new exposure. Bethesda titer of HAα-FVIII(−) patients was monitored for two months after FVIII exposure to verify the production of inhibitors. Patients with human immunodeficiency virus and hepatitis C virus infections and individuals with apparent infections or inflammatory processes were excluded from the study. For all patients, the Bethesda titer was confirmed in a second sample.

**Table 1:** **Characterization of patients with HA who were positive for inhibitors**

**Patient number**	**Age (years)**	**Level of circulating FVIII**	**Severity**	**Historical peak of Bethesda titer (UB/mL)**	**Bethesda titer (UB/mL) at the time of blood collection**
1	11	2.0	Moderate	16.0	16.0
2	19	<1.0	Severe	3.2	0.0
3	35	<1.0	Severe	384.0	20.8
4	11	2.7	Moderate	12.8	8.4
5	14	<1.0	Severe	192.0	20.8
6	13	1.9	Moderate	5.8	5.8
7	40	<1.0	Severe	93.0	16.0
8	19	<1.0	Severe	160.0	12.0
9	27	<1.0	Severe	5.4	5.4
10	6	4.0	Moderate	14.4	6.4
11	37	1.8	Moderate	128.0	0.6
12	22	<1.0	Severe	288.0	115.2
13	31	<1,0	Severe	25.6	14.4
14	14	2.9	Moderate	36.0	5,2
15	8	1.0	Severe	384.0	4.6
16	16	<1.0	Severe	7.0	2.8
17	38	<1.0	Severe	48.0	0.3
18	8	2.8	Moderate	768.0	2.0
19	10	1.0	Severe	48.0	1.2
20	36	1.5	Moderate	13.0	0.0
21	14	<1.0	Severe	52.0	13.2
22	39	<1.0	Severe	3.9	0.0
23	1	1.6	Moderate	6.6	0.5
24	23	<1.0	Severe	224.0	1.4
25	57	<1.0	Severe	20.8	1.0

### Immunophenotyping of cell subsets and intracellular cytokines

Peripheral blood cells were immunostained in the dark for 30 min at room temperature with TriColor-labelled [TC-phycoerythrin (PE)-cyanin 5 (Cy5)] monoclonal antibodies (mAbs) (Caltag, Burlingame, CA, USA), including anti-CD4 (clone S3.5), anti-CD8 (clone M-L233), anti-CD14 (clone TüK4), anti-CD16 (clone 3G8), and anti-CD19 (clone 4G7) mAbs. After lysis/fixation, the leukocytes were permeabilized by incubation with phosphate-buffered saline (PBS) permeabilization reagent (PBS supplemented with 0.5% [w/v] saponin [Sigma, St Louis, MO, USA]) for 10 min at room temperature. Fixed/Permeabilized cells were then incubated in the dark for 30 min at room temperature with 20 μL PE-labeled anti-cytokine mAbs (IFN-γ, clone B27; TNF-α, clone MAB11; IL-4, clone MP4-25D2; IL-5, clone TRFK5; and IL-10, JES3-9D7 [e-Bioscience, San Diego, CA, USA]) in PBS permeabilization reagent.

### Flow cytometry acquisition and analysis

After immunophenotyping, leukocyte suspensions were run in a FACScalibur® flow cytometer (Becton Dickinson, San Jose, CA, USA) to collect 30,000 ungated events per sample. The acquired data were analyzed using CellQuest software (Franklin Lakes, NJ, USA). Distinct gating strategies were used to analyze cytokine-expressing leukocyte subpopulations from innate and adaptive immune cells. Neutrophils were selected as SSC^High^CD16^High+^ cells and monocytes were selected as CD14^High+^ cells on FL3/anti-CD16-TC and FL3/anti-CD14-TC versus laser side-scatter (SSC) dot plots, respectively. Lymphocyte populations were first selected on laser forward-scatter (FSC) versus SSC dot plots. The number of gated neutrophils, monocytes, and lymphocytes ranged from 19,500 to 22,500, 1,200 to 2,100, and 6,000 to 9,000, respectively. After the initial gate selection, the frequencies of cytokine-positive cells were quantified using quadrant statistics applied on FL3/anti-cell surface marker-TC versus FL2/anti-cytokine-PE dot plots. PE-Cy5-labeled antibodies were detected on FL3 channel, and PE-labeled antibodies were detected on FL2 channel. Distinct tubes were used to evaluate the percentage of cytokine-positive T cells (CD4^+^ and CD8^+^) and B cells (CD19^+^). Data were expressed as the percentage of cytokine-positive cells among gated neutrophils, monocytes, and total lymphocytes. The results were assembled further to calculate the global cytokine profiles of immune cells, as proposed earlier [12]. Briefly, the median percentage for each cytokine-positive cell population was calculated using the values obtained for each study group [BD, HAα-FVIII(−), and HAα-FVIII(+) groups].

### Statistical analysis

Statistical analyses were performed using GraphPad Prism 5 software (San Diego, CA, USA). Because all data files assumed a non-Gaussian distribution, statistical comparisons were performed using non-parametric Kruskal–Wallis test followed by Dunn’s multiple comparison test to evaluate cytokine profiles of innate and adaptive immune cells from BDs, HAα-FVIII(−) patients, and HAα-FVIII(+) patients. Differences were considered significant when p-values were <0.05.

## Results

### Monocytes and neutrophils from HAα-FVIII(+) patients have an anti-inflammatory/regulatory cytokine profile

**Figure 1:**
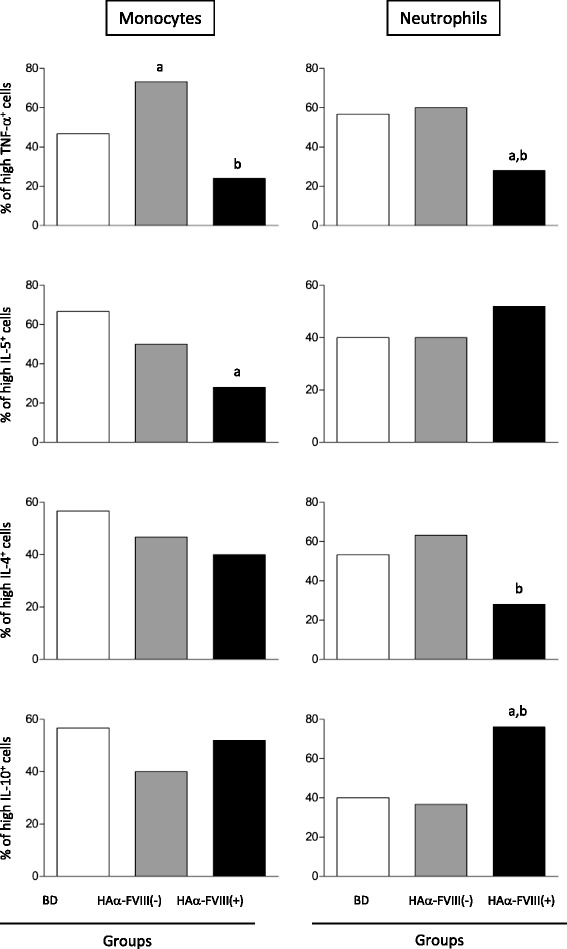
**Analysis of intracytoplasmic cytokine profile of monocytes and neutrophils from the peripheral blood of blood donors (BDs; white bars), patients with HA who are negative for anti-FVIII inhibitors (HAα-FVIII[−]; light gray bars), and patients with HA who are positive for anti-FVIII inhibitors (HAα-FVIII[+]; dark gray bars).** Statistical significance at *p < 0.05* is represented by letters “a”, “b”, and “c” for comparisons with BDs*,* HAα-FVIII(−) patients, and HAα-FVIII(+) patients, respectively. The number of gated neutrophils and monocytes ranged from 19,500 to 22,500 and from 1,200 to 2,100, respectively.

Analysis of monocytes showed significantly lower frequencies of TNF-α-positive and IL-5-positive cells in HAα-FVIII(+) patients than in HAα-FVIII(−) patients and BDs. The analyses also showed considerable frequencies of monocytes with a high production of TNF-α in HAα-FVIII(−) group when compared to the BD group. All the three groups had similar frequencies of IL-4-positive and IL-10-positive monocytes. Analysis of neutrophils showed lower frequency of TNF-α-positive cells in HAα-FVIII(+) patients than in HAα-FVIII(−) patients and BDs. Conversely, higher frequencies of IL-10-positive neutrophils were observed in HAα-FVIII(+) patients than in HAα-FVIII(−) patients and BDs. Moreover, lower frequencies of IL-4-positive neutrophils were observed in HAα-FVIII(+) patients than in HAα-FVIII(−) patients. However, the three groups showed similar frequencies of IL-5-positive neutrophils (Figure 1).

### High synthesis of IL-10 is the hallmark of CD4^+^ and CD8^+^ T cells in HAα-FVIII(+) patients

**Figure 2:**
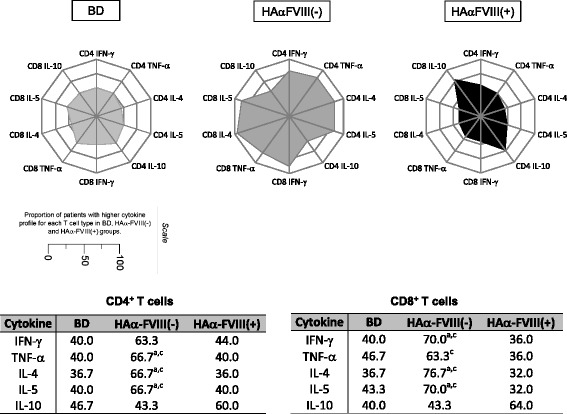
**Overall proinflammatory and anti-inflammatory/regulatory cytokine patterns of lymphocytes. (a)** Radar chart summarizes the percentage of proinflammatory and anti-inflammatory/regulatory cytokine balance in adaptive immune cells from BDs (light gray area), HAα-FVIII(−) patients (dark gray area), and HAα-FVIII(+) patients (black area). Each axis displays the proportion of each cytokine balance category within a given leukocyte subset. **(b)** Median percentage of each cytokine T cell population studied for groups BD, HAα-FVIII(−) and HAα-FVIII(+). Statistical significance at *p < 0.05* is represented by letters “a”, “b”, and “c” for comparisons with BDs, HAα-FVIII(−) patients, and HAα-FVIII(+) patients, respectively.

Immunophenotyping of adaptive immune cells showed that T cells (CD4^+^ and CD8^+^) of BDs had basal levels of all the analyzed cytokines. Furthermore, T cells of HAα-FVIII(−) patients had significantly elevated levels of all the cytokines, except IL-10. However, T cells of HAα-FVIII(+) patients only had elevated levels of IL-10 (Figure 2).

### B cells from patients with HA and BDs have similar cytokine profiles

**Figure 3:**
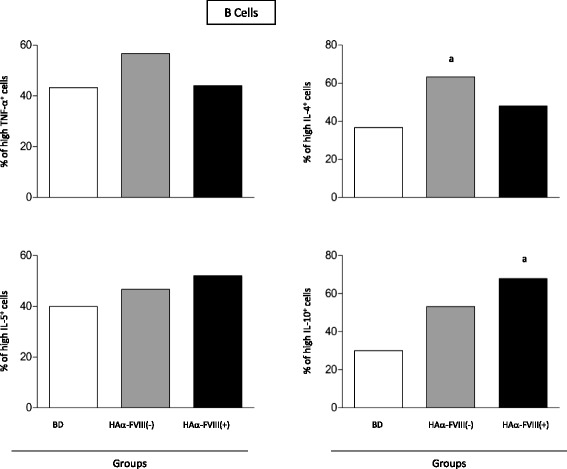
**Percentage of TNF-α-positive, IL-5-positive, IL-4-positive, and IL-10-positive B cells from the peripheral blood of BDs (white bars), HAα-FVIII(−) patients (light gray bars), and HAα-FVIII(+) patients (dark gray bars).** Statistical significance at *p < 0.05* is represented by letters “a”’, “b” and “c” for comparisons with BDs*,* HAα-FVIII(−) patients, and HAα-FVIII(+) patients, respectively.

Analysis of B cells showed higher levels of IL-4-positive cells in HAα-FVIII(−) patients than in BDs. In addition, higher frequency of IL-10-positive B cells was observed in HAα-FVIII(+) patients than in BDs. However, the three groups showed similar frequencies of TNF-α-positive and IL-5-positive B cells (Figure 3).

## Discussion

The treatment regimen of FVIII varies among different countries and among centers in the same country. For patients included in this study, on-demand treatment was used to manage clinically evident bleeding. FVIII replacement therapy depends on age, severity, and treatment regimen. Recent studies have shown that median annual on-demand FVIII utilization (IU kg^−1^ · year^−1^) varies between 1,100 and 1,429 IU kg^−1^ · year^−1^ [13,14]. Our data showed that patients receiving on-demand treatment at Fundação Hemominas used 1,452 IU kg^−1^ · year^−1^ of FVIII. Recent reports have shown that replacement therapy with these amounts of FVIII products can cause several changes in the immune profile of patients with HA [15-19].

Production of anti-FVIII inhibitors remains a challenge in the treatment of patients with HA. Therefore, understanding the cellular compartmentalization of immune responses is important. Little is known about the cytokine profiles and cell types implicated in auto- and alloimmune responses to FVIII. Previous studies have shown that patients with HA who are positive for inhibitors have a major anti-inflammatory/regulatory immune cytokine profile while those without inhibitors have a mixed pattern, with a bias toward an inflammatory cytokine profile. These findings support and suggest that proinflammatory-modulated immune response may favor the synthesis of anti-FVIII IgG1 antibodies and prevent the synthesis of anti-FVIII IgG4 inhibitors [12,20,21]. It has been suggested that immunological context along with the intensity of treatment favors the class switching of FVIII-specific antibodies to IgG4 [22].

The findings of the present study highlight the significantly lower frequencies of TNF-α-positive monocytes and neutrophils, IL-5-positive monocytes, and IL-4-positive neutrophils and higher frequencies of IL-10-positive neutrophils in HAα-FVIII(+) patients. Recent studies have shown that neutrophils not only synthesize cytokines in response to various inflammatory stimuli in chronic inflammatory disorders (such as rheumatoid arthritis, inflammatory bowel diseases, diabetes, and mycobacterial infections) but also regulate immune responses such as those suggested in HA. Some studies have also proposed that the cytokine profile of neutrophils is similar to the cytokine profiles of monocytes and macrophages. Therefore, modulation of cytokines derived from monocytes and neutrophils is a potentially useful strategy for therapeutic immunointervention [23-25].

Stimulation of B cells to produce FVIII inhibitors is dependent on CD4^+^ T [26]. However, recent reports describe the contribution of CD8^+^ T cells in this context. A recent study has shown an association between CD8^+^ T cells and induction of immune tolerance. It was found that human CD8^+^ T cells also functioned as regulatory T cells to induce immune tolerance by suppressing activated T cells through IL-10 production [27-29]. In addition, CD8^+^ cytotoxic cells mediate the apoptosis of T cells that contribute to peripheral tolerance [30]. Proliferation of CD8^+^ suppressor cells and activated CD8^+^ T cells is observed in patients with HA; moreover, proliferation of suppressor cells seems to be mainly related to FVIII infusion [31]. These regulatory T cells are important for regulating other T cells without the intervention of antigen-presenting cells (APCs). In addition, it is well known that repeated infusions of pdFVIII concentrates skews TCR repertoires for CD8^+^ T cells [15,19].

Analysis of T cells from HAα-FVIII(−) patients showed elevated levels of almost all cytokines. In contrast, T cells from HAα-FVIII(+) patients showed decreased levels of all the tested cytokines, except IL-10. The immune response against FVIII develops as a classical T cell-dependent antibody-mediated immune response. Infused FVIII is recognized, internalized, and processed by APCs and is presented to antigen-specific CD4^+^ T cells that provide activation signals to antigen-specific B cells, thus resulting in the synthesis of anti-FVIII antibodies and long-lived FVIII-specific memory B cells [8,32,33]. The data obtained in this study showed that the cytokine microenvironment promoted by T cells is determinant for the presence or absence of FVIII inhibitors. These data are consistent with those of different studies that observed a mixed cytokine pattern, with a bias toward an inflammatory cytokine profile, in HAα-FVIII(−) patients and anti-inflammatory/regulatory pattern in HAα-FVIII(+) patients [7,8,12,34]. The changes observed in CD8^+^ T cell compartment also seem to be related to the production of inhibitors and the regulatory profiles of these cells. In HAα-FVIII(−) patients, increased cytokine production by lymphocytes contributed to the prevention of inhibitor production by modulation of the immune system; in contrast, in the inhibitor-positive patients, reducing the production of cytokines inducing immune microenvironment modifying the expansion of lymphocytes with a regular profile, increasing the production of IL-10 in order to re-modulate the immune system; However, other changes, which need investigation, prevent this process, thus resulting in the production of inhibitors.

Analysis of B cells showed that HAα-FVIII(−) patients had higher levels of IL-4-positive B cells and HAα-FVIII(+) patients had higher levels of IL-10-positive B cells. Because of their central role in inhibitor production, it is important to elucidate the cytokine profile of B cells in patients with HA. The IL-10-predominant cytokine profile of B cells observed in this study is well characterized as a downregulator of TNF-α, IL-1α, and IL-1β production. However, it stimulates B cell proliferation and differentiation and antibody production [35]. Importantly, B cells are not merely antibody producers but actively contribute by regulating immune response and secreting cytokines that amplify humoral and cellular immune responses [36-39].

## Conclusions

Typically, various cytokines and chemokines act as mediators in an immune response. It is important to note that genes encoding IL-10 and TNF-α were the first genes located outside the gene encoding FVIII to be associated with inhibitor production in patients with HA [9-11,40]. However, different risk factors such as ethnicity, type of mutation in the gene encoding FVIII, family history of inhibitor production, type of FVIII (plasmatic or recombinant) therapy, patient age at the time of first exposure to replacement therapy, initial doses of FVIII concentrate, mode of infusion, surgery, intensity of treatment or regular prophylaxis, and inflammatory state or HLA haplotype of patients are associated with inhibitor production and should be investigated further. Other causes of inhibitor production against FVIII include stress, age, malignancy, coinfections, pregnancy, and antibiotics [41-43]. Based on these findings, we hypothesize that anti-inflammatory/regulatory cytokine-dominant microenvironment determined by monocytes, neutrophils, T cells, and B cells may favor the synthesis of anti-FVIII IgG4 with inhibitory activity. In addition, the proinflammatory microenvironment corroborates with the synthesis of anti-FVIII IgG1 non-inhibitory antibody [12,20,21].

The limitations of the study include limited number of patients and absence of information on mutations in the gene encoding FVIII. An important issue to be considered in future investigations is to analyze the kinetic conversion of a proinflammatory immune response to an anti-inflammatory/regulatory immune response against FVIII in HAα-FVIII(+) patients and the risk factors that may be associated with this conversion during HA treatment. The anti-inflammatory/regulatory cytokine profile of innate immune cells in patients with HA could be an important biomarker for the development of high inhibitor titers [7].
